# Direct repair of lumbar spondylolysis by Buck’s technique

**DOI:** 10.4103/0019-5413.77133

**Published:** 2011

**Authors:** S Rajasekaran, M Subbiah, Ajoy Prasad Shetty

**Affiliations:** Department of Orthopedics and Spine Surgery, Ganga Hospital, Coimbatore, Tamil Nadu, India

**Keywords:** Buck’s technique, direct pars repair, pars interarticularis, spondylolysis

## Abstract

**Background:**

The lesion in spondylolysis is a nonunion that follows a fatigue fracture of pars interarticularis. Direct repair of the pars defect is a logical alternative to fusion as it helps to preserve the motion segment and prevents abnormal stresses at the adjacent levels. The purpose of the study is to analyze the clinical and radiological results of direct screw osteosynthesis of the pars defect by the Buck’s method in patients with symptomatic spondylolysis with or without grade 1 spondylolisthesis.

**Materials and Methods:**

Nine patients (six males, three females, mean age 24 years) with symptomatic spondylolysis with or without grade 1 spondylolisthesis and a normal disc in magnetic resonance imaging (MRI), who failed conservative treatment, underwent surgery between January 2000 and April 2009. Of them five patients had bilateral lysis at one level, one had bilateral lysis at three levels and two levels each and two had unilateral lysis at one level. Direct pars repair by the Buck’s method with internal fixation of the defect using 4.5 mm cortical screws and cancellous bone grafting was done. The mean follow-up period was 45 months. MacNab criteria were used to evaluate the postoperative functional outcome. Healing of the pars defect was assessed by plain radiographs and computed tomography (CT) scan.

**Results:**

Spondylolysis was bilateral in seven and unilateral in two patients. Two patients had associated grade 1 spondylolisthesis. The mean operative time was 58 minutes (range 45 – 75 minutes) and blood loss was 98 ml (50 – 140 ml). Although radiological fusion was observed in all patients at a mean follow-up of 45 months (range 9 to 108 months), the functional outcome was excellent in two patients and good in five, with one fair and one poor result. The overall result of the procedure was satisfactory in 78% (7/9) of the patients. The two patients with associated grade 1 spondylolisthesis had fair and poor results. No complications were encountered in the perioperative or postoperative period.

**Conclusions:**

In carefully selected patients, direct repair of the pars defect by the Buck’s technique of internal fixation and bone grafting was a safe and effective alternative to fusion in younger patients with symptomatic spondylolysis, without associated spondylolisthesis, who failed conservative management.

## INTRODUCTION

Lumbar spondylolysis is a nonunion following a stress fracture of the pars interarticularis.^1^,^2^ It is a common cause of low back pain in children, adolescents, and young adults,^3^ with an incidence of 3 – 10% in the general population and 6% in adults.^4^ A majority of these patients respond to conservative treatment.^5^ Surgical intervention is indicated in patients with persistent pain, unresponsive to conservative management.^6^,^7^ Surgical procedures that have been described earlier for this entity are posterior and posterolateral fusion.^8^ The disadvantage of these procedures is loss of one motion segment of the lumbar spine, with an increased risk of adjacent level degeneration.^9^ As the lesion in spondylolysis is an acquired pseudarthrosis, direct repair of the defect with internal fixation and cancellous bone grafting will be a logical treatment option that obviates the need for an arthrodesis of that motion segment. Buck’s technique of pars repair with screw fixation and bone grafting of the defect is a viable alternative to spinal fusion in selected patients of spondylolysis.^2^

The purpose of this study is to review results of the direct screw osteosynthesis by the Buck’s technique, in nine cases of lumbar spondylolysis, with or without grade 1 spondylolisthesis.

## MATERIALS AND METHODS

Between January 2000 and April 2009, nine patients with symptomatic lumbar spondylolysis were treated using the Buck’s technique of direct pars repair and all these patients were included in the study. Six of the patients were male and three were female. All the patients had low back pain without radicular symptoms. The mean duration of back pain before surgery was 11 months (range: 6 – 24 months). The preoperative physical examination was normal, apart from the decreased range of forward flexion.

Plain anteroposterior and lateral radiographs and CT scans were obtained for all the patients. Five patients had bilateral lysis at one level, one had bilateral lysis at three levels, one had bilateral lysis at two levels and two had unilateral lysis at one level. Seven patients had spondylolysis of L5. One patient had spondylolysis at three levels involving L2, L3, and L5, and one had a double level defect involving L4 and L5. Two patients had grade 1 lytic spondylolisthesis of L5 – S1. (MRI) was obtained for all the patients, and the presence of a normal disc without any degenerative changes of the disc or facet joint, at the level of spondylolysis, was confirmed in the T2 weighted images.

All the patients underwent an adequate trial of conservative treatment including rest, analgesics, abdominal strengthening exercises, and antilordotic bracing for a period of six months, without benefit. Patients with the following criteria were selected as candidates for pars repair by Buck’s technique: radiologically confirmed spondylolysis with or without grade 1 spondylolisthesis, with chronic disabling low back pain, unresponsive to conservative treatment for six months, with a normal disc and facet joint at the level of spondylolysis, which was confirmed by MRI.

### Operative procedure

The surgical technique was as described by Buck.^2^,^10^ After a standard posterior midline exposure of the involved vertebra, the fibrous tissue and the sclerotic bone in the lytic defect was removed and prepared till bleeding bony surface. The entry point was made by creating a notch in the caudal margin of the lamina, 10 mm lateral to the base of the spinous process. A 3.2 mm drill-bit was used to drill the path of the screw with the trajectory angled 30° lateral to the sagittal plane, toward the ipsilateral pedicle, crossing the lytic defect. An appropriately sized 4.5 mm titanium cortical screw was inserted along the path across the defect, but not tightened completely. The cancellous bone grafts obtained from the posterior iliac crest were packed in the lytic defect and the screw was tightened completely to obtain a good purchase in the solid bone of the ipsilateral pedicle.

The patients were allowed to sit and ambulate with a lumbosacral orthosis on the first postoperative day. The corset was worn for a period of six weeks. The patients were reviewed at 1, 3, 6, 9, and 12 months. The radiological fusion of the defect was assessed by plain radiographs or a CT scan. The MacNab criteria^11^ [Table 1] were used to assess the postoperative clinical outcome.

**Table 1:** MacNab criteria^11^

Grade	Description	
Excellent	No pain	Full activity with work
Good	Occasional pain	Not interfering with work
Fair	Pain occasionally	Interfering with work
Poor	Persistent pain	Frequently interfering with work

## RESULTS

The mean age at the time of surgery was 24 years (range: 15 – 31 years). The mean operative time was 58 minutes (range: 45 – 75 minutes), and the mean blood loss was 98 ml (50 – 140 ml). There was no postoperative neurological deficit or wound infection. The mean follow-up period was 45 months (range: 9 – 108 months).

Two patients were able to return to their former occupation without pain and they were considered to have an excellent outcome. Five patients had occasional mild back pain, but were able to resume their former occupation, and these results were rated as a good outcome. Thus, an overall satisfactory result with excellent and good outcome was observed in 78% (7 / 9) of the patients. One patient had continued low back pain that was less than the preoperative pain, but interfered with the patient’s work. Hence the patient had to change the occupation to less strenuous activities. This patient was rated to have a fair outcome. One patient had persistent back pain similar to that of preoperative pain and was unable to return to his former occupation. He was seeking continuous medical care and was on regular analgesics. He was rated to have a poor outcome. Although the healing was complete in this case, his follow-up MRI revealed a grey disc with hypointensities within the nucleus pulposus, suggestive of disc degeneration.

**Figure 1:**
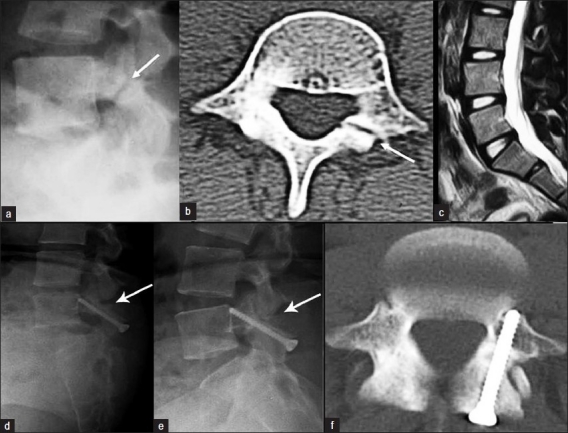
(a) Preoperative lateral radiograph and (b) axial CT scan showing unilateral defect of the pars interarticularis of the L4 vertebra. (c) Sagittal T2 weighted MRI demonstrating the normal L4-L5 disc without any degeneration. Follow-up lateral dynamic radiographs in (d) flexion and (e) extension, showing complete healing of the defect without signs of instability. (f) Postoperative axial CT scan demonstrating complete healing of the spondylolytic defect

**Figure 2:**
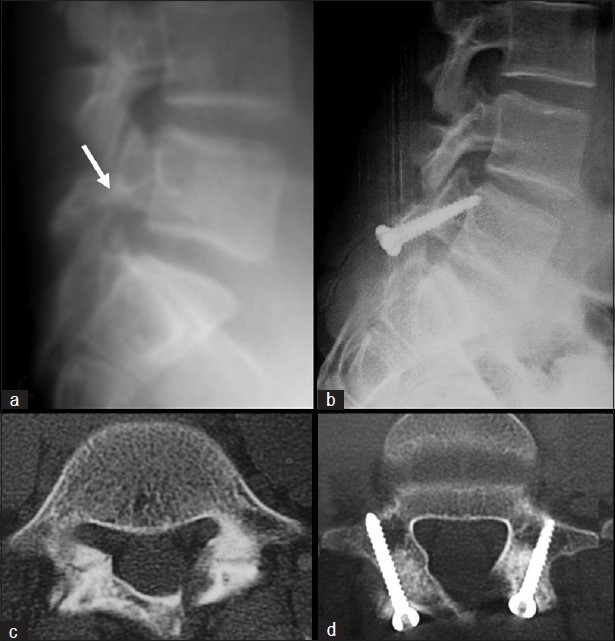
(a) Preoperative lateral radiograph and (b) follow-up lateral radiograph of a patient with bilateral spondylolysis of L5 fixed by Buck’s technique on both sides. (c) Preoperative and (d) follow-up axial CT scan of the same patient showing complete healing of the pars defect bilaterally, without signs of screw loosening, back out, or breakage

Bony fusion was assessed with the help of plain radiographs, and in doubtful cases CT scans were obtained. Complete radiographic healing of the spondylolytic defect was seen in all patients [Figures 1 and 2]. There was no screw breakage or back out.

## DISCUSSION

The pathological lesion in spondylolysis is a nonunion following a fatigue fracture in the pars interarticularis.^1^,^2^,^12^ The incidence of lumbar spondylolysis is between 3 – 10%, in the general population.^4^ It is a common cause of low back pain in children and young adults. Spondylolysis is frequently encountered in adolescents, as they indulge in strenuous activities when their intervertebral discs are more elastic and the neural arches are not completely ossified.^13^ The postulated causes of pain in spondylolysis include the rich nociceptive nerve endings within the defect, hypermobility of the loose posterior arch with stimulation of the nerve endings within the defect, relative instability of the vertebral body, and excessive stress on the underlying disc.^14^–^16^

A majority of these patients respond well to conservative management.^4^ Surgical intervention is indicated only in patients with persistent pain unresponsive to adequate trial of conservative treatment.^17^,^18^ The surgical procedures that have been described earlier involve arthrodesis of the motion segment by posterolateral^19^ or interbody fusion techniques.^20^ These procedures sacrifice the mobility of the involved motion segment and increase the mechanical stress in the adjacent level,^9^ both of which are deleterious in this younger age group. As the lesion in spondylolysis is an acquired pseudarthrosis, the logical treatment would involve internal fixation and bone grafting of the defect, as in the case of nonunion of a long bone.

**Figure 3:**
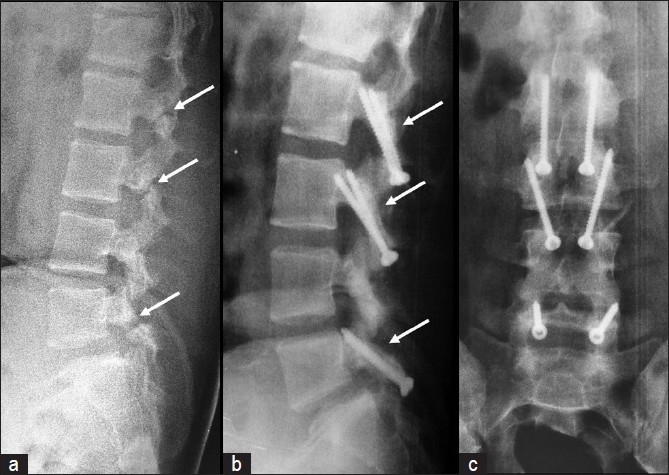
(a) Preoperative lateral radiograph of a patient with triple level spondylolysis at L2, L3, and L5. (b) Postoperative follow-up lateral and (c) anteroposterior radiographs showing good healing of the pars defect at all three levels

Various procedures have been described for the direct repair of spondylolysis. Kimura in 1968,^21^ first described pars repair with isolated bone grafting of the defect, without internal fixation, and he used a postoperative cast for immobilization. Buck in 1970, first used a screw to stabilize the repair, in addition to bone grafting, and reported one failure and two complications in his series of 16 patients.^2^ Nicol and Scott^22^ used tension-band wiring and Morscher^23^ used a hook screw for fixation of pars defects. Gillet and Petit^16^ used a V-shaped rod-pedicle screw construct for pars fixation in 10 patients and reported satisfactory results in 70% of the patients. Deguchi **et al**.^24^ made a biomechanical comparison of various pars fixation procedures including, Buck’s technique, Scott’s technique, and the screw-rod-hook fixation technique, and concluded that the Buck’s technique resisted forces and moments in any direction. This technique was indicated for patients less than 30 years of age, pars defects less than three or four millimeters^2^, with or without grade 1 spondylolisthesis,^7^,^25^ with a normal disc and facet joint confirmed by MRI. An overall satisfactory result with an excellent or good clinical outcome was observed in seven patients (78%) in our series. The clinical outcome was good in patients with double and triple level spondylolysis, in whom the procedure was done at all the involved levels [Figure 3]. Radiological fusion was observed in all our patients. The success rate of Buck’s technique reported in the literature ranged from 67 to 93%.^7^ Askar **et al**.^26^ reported a success rate of 85% and a fusion rate of 100% in 14 patients who underwent pars repair by the Scott’s technique. However, larger dissection was needed to insert wires under the transverse process and there was an increased risk for nerve root injury and excessive bleeding during the Scott’s procedure.^8^,^16^ Gillet **et al**.^16^ reported 70% satisfactory result, with fusion occurring in all patients treated with a V-shaped rod-pedicle screw construct. Ivanic **et al**.^27^ reported a pseudarthrosis rate of 13.3% following the use of Morscher’s hook screw for stabilizing the pars defect. Definitive assessment of a bony union is difficult with plain radiographs alone. CT scans, for confirmation of healing of the lesions, were conducted only in those patients who were symptomatic during their follow-up evaluation, as described by Debnath **et al**.^28^ One patient had a fair result with minimal low back pain, activity restriction, and diminished range of flexion. He had grade 1 spondylolisthesis of the L5 – S1 on the preoperative radiographs, but complete radiological fusion of the defect was observed in this case. One patient had a poor result despite complete healing of the defect with persistent back pain, similar to preoperative pain, and an inability to return to his former occupation. This patient also had grade 1 spondylolisthesis of L5-S1. Repeat MRI of the lumbar spine in this patient revealed a gray disc at L5-S1 with hypointensities within the nucleus pulposus, suggestive of disc degeneration. He was advised fusion of L5-S1, but the patient refused to undergo the procedure. The causes of failure described by Buck in his series of patients include improper screw placement across the defect and inadequate purchase of the screw in the pedicle.^2^ The fair and poor results in our patients were seen in cases of spondylolysis with associated grade 1 spondylolisthesis. We speculate that, the source of symptoms in these cases was probably due to the ongoing degenerative changes in the underlying disc, secondary to minimal pre-existent spondylolisthesis. Thus, even though Buck’s technique was described in patients with grade 1 spondylolisthesis,^25^,^29^ we believe that this procedure should be limited to patients with spondylolysis without associated spondylolisthesis, to obtain a satisfactory clinical outcome.

The advantages of direct pars repair by Buck’s technique include: restoration of normal anatomy of the posterior elements, preservation of the functional motion segment in this group of young patients [Figure 3], less surgical trauma with dissection restricted only medial to the facet joint, less blood loss, and early functional recovery. However, careful patient selection is very important for the success of this procedure. The disc and facet joint at the involved level should be completely normal, without any signs of degeneration in the MRI and the technique should preferably be limited to patients with spondylolysis, without associated spondylolisthesis.

## CONCLUSION

In carefully selected patients, direct repair of the pars defect by Buck’s technique of internal fixation and bone grafting is a safe and effective alternative to fusion in younger patients with symptomatic spondylolysis, without associated spondylolisthesis, who fail conservative management.
